# Antidepressant Low Doses of Ketamine and Melatonin in Combination Produce Additive Neurogenesis in Human Olfactory Neuronal Precursors

**DOI:** 10.3390/molecules27175650

**Published:** 2022-09-01

**Authors:** Rosa Estrada-Reyes, Daniel B. Quero-Chávez, Salvador Alarcón-Elizalde, Montserrat G. Cercós, Citlali Trueta, Luis A. Constantino-Jonapa, Julián Oikawa-Sala, Jesús Argueta, Ricardo Cruz-Garduño, Margarita L. Dubocovich, Gloria A. Benítez-King

**Affiliations:** 1 Laboratorio de Fitofarmacología, Dirección de Investigaciones en Neurociencias, Instituto Nacional Psiquiatría Ramón de la Fuente Muñiz, Calzada Mexico-Xochimilco 101, San Lorenzo Huipulco, Tlalpan, Ciudad de México 14370, Mexico; 2 Laboratorio de Neurofarmacología, Subdirección de Investigaciones Clínicas, Instituto Nacional de Psiquiatría Ramón de la Fuente Muñiz, Calzada Mexico-Xochimilco 101, San Lorenzo Huipulco, Tlalpan, Ciudad de México 14370, Mexico; 3 Departamento de Neurofisiología, Dirección de Investigaciones en Neurociencias, Instituto Nacional de Psiquiatría Ramón de la Fuente Muñiz, Calzada México-Xochimilco 101, San Lorenzo Huipulco, Tlalpan, Ciudad de México 14370, Mexico; 4 Department of Pharmacology and Toxicology, Jacobs School of Medicine and Biomedical Sciences, University at Buffalo (SUNY), 955 Main Street, Buffalo, NY 14203, USA

**Keywords:** melatonin, ketamine, neurogenesis, olfactory neuronal precursors, antidepressants

## Abstract

Melatonin (MEL), an indolamine with diverse functions in the brain, has been shown to produce antidepressant-like effects, presumably through stimulating neurogenesis. We recently showed that the combination of MEL with ketamine (KET), an NMDA receptor antagonist, has robust antidepressant-like effects in mice, at doses that, by themselves, are non-effective and have no adverse effects. Here, we show that the KET/MEL combination increases neurogenesis in a clone derived from human olfactory neuronal precursors, a translational pre-clinical model for effects in the human CNS. Neurogenesis was assessed by the formation of cell clusters > 50 µm in diameter, positively stained for nestin, doublecortin, BrdU and Ki67, markers of progenitor cells, neurogenesis, and proliferation. FGF, EGF and BDNF growth factors increased the number of cell clusters in cultured, cloned ONPs. Similarly, KET or MEL increased the number of clusters in a dose-dependent manner. The KET/MEL combination further increased the formation of clusters, with a maximal effect obtained after a triple administration schedule. Our results show that the combination of KET/MEL, at subeffective doses that do not produce adverse effects, stimulate neurogenesis in human neuronal precursors. Moreover, the mechanism by which the combination elicits neurogenesis is meditated by melatonin receptors, CaM Kinase II and CaM antagonism. This could have clinical advantages for the fast treatment of depression.

## 1. Introduction

Melatonin (*N*-acetyl-5-methoxytriptamine; MEL) is an ancient, phylogenetically well-conserved molecule [1]. This indolamine is synthesized in the pineal gland in accordance with the photoperiod [1] and directly released into the cerebrospinal fluid and into general circulation [2,3]. Additionally, MEL is synthesized in non-pineal tissues. Among these, melatonin is produced in the brain in specific regions such as the cerebellum [4].

Pineal and local synthesis of MEL can be responsible for the high levels of this indolamine observed in the brain. The precise physiological meaning of high levels of MEL in the CNS is not known, but presumably, it is to protect neurons and glia from oxidative stress and inflammation, as well as to stimulate the formation of new neurons in specific niches [5]. In addition, MEL in the CNS has antidepressant-like effects, associated with neurogenesis and dendritogenesis produced in the dentate gyrus of the hippocampus of rodents [6,7,8]. Similar effects have been demonstrated for ISSR antidepressants in animal models and postulated as a therapeutic mechanism in humans [8].

Recently, we described that administration of MEL at non-effective antidepressant doses, in combination with non-effective psychomimetic doses of ketamine (KET), a *N*-methyl-*D*-aspartate (NMDA) receptor antagonist under single or triple administration protocols, causes antidepressant-like effects in mice associated with increased neurogenesis in the dentate gyrus of the hippocampus, without psychomimetic effects [8].

Despite animal models being very useful in the characterization of antidepressant drugs, studies in humans are necessary, because sometimes the effects observed in animals are not reproduced. Clinical trials are expensive and complex, and therefore, pre-clinical human neuronal models that reflect biological and biochemical responses observed in the CNS are necessary to translate pharmacological responses to humans before starting clinical trials.

In the last two decades, the neuroepithelial tissue, as well as olfactory neuronal precursors (ONPs) derived from it, have been used as experimental models to study the etiology of neuropsychiatric diseases and as a model of neurodevelopment [9,10,11,12]. Pharmacological responses also have been studied in these cells to validate drug responses for the treatment of Parkinson’s disease [13]. Neuronal precursors derived from this tissue and cultured in selective media [14,15] been used to characterize schizophrenia, bipolar disorder and Alzheimer’s disease biomarkers [16,17,18].

The neuroepithelial tissue is formed by sustentacular cells, olfactory neurons and globose cells which are stem cells that, in culture, actively proliferate, forming in a first stage adhered clusters stained with anti-nestin, anti-BrdU, anti-pheripherin and anti-*β*III tubulin antibodies [19]. In a later stage, these clusters grow and increase in size and then are detached from the surface of the Petri dish, floating in the culture media with a structure of neurospheres, indicating that these cells retain their neurogenic properties [14,20]. Similarly, cloned cells derived from these cultures also form these clusters and neurospheres [10]. 

The embryologic neuronal linage of the neuroepithelium is similar to the limbic region, with gene and neurotransmitter´s receptor expression (NMDA, serotonin, melatonin, brain-derived neurotrophic factor (BDNF), and dopamine) similar to those produced in neurons of the hippocampus, one of the main target of antidepressants currently used in the clinic [21,22]. The capability of globose cells to generate new cells of neuronal linage, as well as the expression of neurotransmitter receptors which are targets for antidepressant drugs in the CNS, makes primary cultures derived from the human olfactory neurepithelium a useful translational model for the screening of new drugs with potential use in the treatment of depression [23].

Since KET/MEL combination produced antidepressant-like effects and increased neurogenesis in mice, we hypothesized that this combination will be able to induce a neurogenic response in a clone of human olfactory neuronal precursors similar to nerve growth factors that have both antidepressant and neurogenic effects in culture and in mice hippocampus [24,25,26,27]. Here, we show that the KET/MEL combination at concentrations and administration schedules similar to those shown to produce antidepressant-like effects induced the formation of spheric clusters positive for neurogenesis markers similarly to the growth factors, fibroblastic growth factor (FGF), epidermal growth factor (EGF) and BDNF. The results also suggest that the neurogenic response elicited by a KET/MEL combination is mediated by melatonin receptors, CaM kinase II and CaM antagonism. The results also indicate that human olfactory neuronal precursors are useful as translational models to characterize the biological effects of drugs used in the treatment of neuropsychiatric diseases.

## 2. Results

### 2.1. Growth Factors Induce Cluster Formation in Human Olfactory Neuronal Precursors in Culture

**Figure 1:**
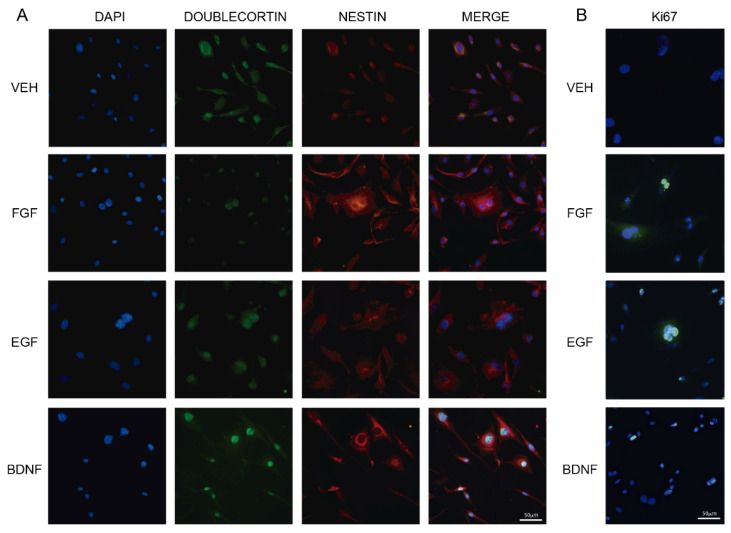
Growth factors elicits cluster formation in human olfactory neuronal precursors. ONPs were incubated during 6 h with 20 ng/mL of either fibroblastic growth factor (FGF), epidermal growth factor (EFG), brain-derived neurotrophic factor (BDNF) or the vehicle (VEH). Then, cells were fixed and stained with anti-doublecortin and anti-nestin antibodies, followed by secondary antibodies coupled to FITC (green) and RITC (red), respectively. Representative images are shown in Panel **A**. Another group of cells was stained with DAPI and an anti-Ki67 antibody, followed by a secondary antibody that recognizes the anti-Ki67 antibody (Panel **B**). Scale bar = 50 µm.

Olfactory neuronal precursors proliferate in culture and form spherical clusters that express nestin [14]. These cells are mitotically active and incorporate BrdU [14]. On the other hand, EFG, FGF, and BDNF stimulate neurogenesis in cultured hippocampal stem cells as well as in the dentate gyrus of the hippocampus in mice [24,25,26,27]. Thus, we tested whether the effects of neuronal growth factors on neurogenesis observed in the mice hippocampus were also elicited in cultured ONPs. For this, we incubated the ONPs with the VEH, or 20 ng/mL of either FGF, EFG, or BDNF, during 6 h. Cell cultures were then stained with antibodies raised against nestin and doublecortin, which are markers of progenitor cells and neurogenesis, respectively [28,29]. Figure 1A shows that ONPs incubated with FGF, EFG, or BDNF formed clusters with a diameter larger than 50 µm. In contrast, a single cell has 10 µm in diameter. Clusters observed in cultures incubated with FGF, EGF, or BDNF were also positive for anti-Ki67 antibody staining, which labels the nuclei of proliferating cells (Figure 1B). Some clusters were not stained with the anti-Ki67 antibody (see FGF Panel). Data suggest that FGF, EGF, and BDNF stimulate neurogenesis in cultured ONPs.

### 2.2. Concentration–Response Effects of Ketamine and Melatonin on Cluster Formation

**Figure 2:**
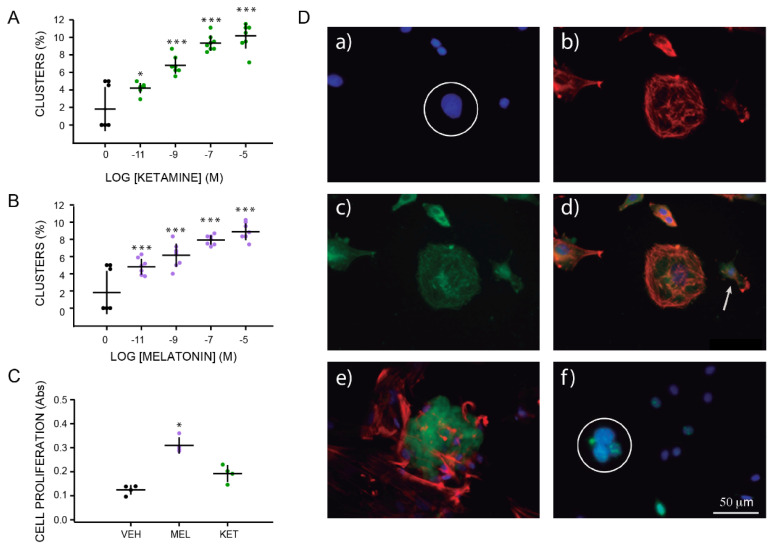
Characterization of clusters formed by olfactory neuronal precursors cultured with either ketamine or melatonin. Graph of the frequency of cluster formation after 6 h of incubation with increasing concentrations of either KET (10^−11^ to 10^−5^ M) or MEL (10^−11^ to 10^−5^ M) are shown in Panels **A**, and **B**, respectively. Formation of clusters was evaluated by counting them in each of 10 chosen fields (n = 10) and normalized by the total number of cells per field determined by DAPI staining. Cell proliferation induced by treatment with vehicle (VEH), MEL (10^−7^ M), or KET (10^−7^ M) were measured by the WST1 transformation by mitochondrial enzymes to formazan and shown in Panel **C**. Each point represents the mean of absorbance values obtained in 4 wells. The mean ± standard deviation of the mean (SEM) is from 1 or 2 experiments performed by quadruplicate. Each experiment was repeated three times. Data were analyzed by one-way ANOVA with Bonferroni’s post-test. * *p* = 0.009; *** *p* = 0.001 when compared with vehicle (VEH) control. Cell proliferation was analyzed by Kruskal–Wallis followed by Dunn´s post-test *p* = 0.001. Panel **D** shows immunofluorescent labeling of ONPs cultured with KET 10^−7^ M during 48 h and stained with DAPI (**a**), phalloidin-TRITC (**b**), and anti-nestin antibody followed by a secondary antibody coupled to FITC (**c**). Merged images show colocalization of these labels (orange) in a spherical cluster (**d**). Image **e** shows a merged image of a spherical cluster simultaneously labeled with an anti-doublecortin antibody followed by a secondary antibody coupled to FITC (green) and phalloidin-TRITC (red). A representative image of clusters stained with DAPI (blue), pre-labeled with BrdU and stained with a specific anti-BrdU antibody (green) is shown in image **f**. Spherical clusters are noted by a white circle in images **a** and **f**. A single cell is noted in **d** with an arrow. Bar = 50 µm.

We tested the effects of either KET or MEL at different concentrations on cluster formation. Figure 2A shows an increased formation of spherical clusters in a concentration–response fashion in the presence of KET or MEL (Figure 2B). Significant differences were observed with an F_(4,35)_ = 46.50, *p* ≤ 0.001, and F_(4,35)_= 30.10, *p* ≤ 0.001, respectively. At 10^−5^ M of either MEL or KET, we observed a nearly four-fold increase in the formation of spherical aggregates (Figure 2A,2B). Cellular growth was assessed as the amount of formazan formed by the cleavage of WST1 by mitochondrial enzymes in the presence of either KET or MEL (Figure 2C). A graph analysis shows that the amount of formazan made in the presence of MEL was 2.5 times that formed in the VEH-incubated cells (Figure 2C), a lower response was observed in the presence of KET, and formazan formed was increased 0.75 times regarding the vehicle-incubated cells (Figure 2C (H = 9.84, df = 2, *p* ≤ 0.001).

Representative images of clusters formed in the ONPs cultures in the presence of KET are shown in Figure 2D. DAPI staining showed the nuclei aggregated (Figure 2Da) phalloidin-TRITC stained the microfilaments (Figure 2Db). Clusters were stained with the anti-nestin and the anti-doublecortin antibodies (Figure 2Dc), while DNA was stained with the anti-BrdU antibody (Figure 2De,f). Merged images show the size of single cells in comparison with the spherical clusters (Figure 2Dd). The anti-doublecortin antibody stained the spherical clusters, while the anti-BrdU antibody stain was observed in the nucleus (Figure 2De,f). These data indicate that either MEL or KET alone stimulate cluster formation in ONPs and that these clusters are formed by proliferating non-differentiated and neurogenic cells.

### 2.3. Ketamine and Melatonin Combination Enhances Cluster Formation

**Figure 3:**
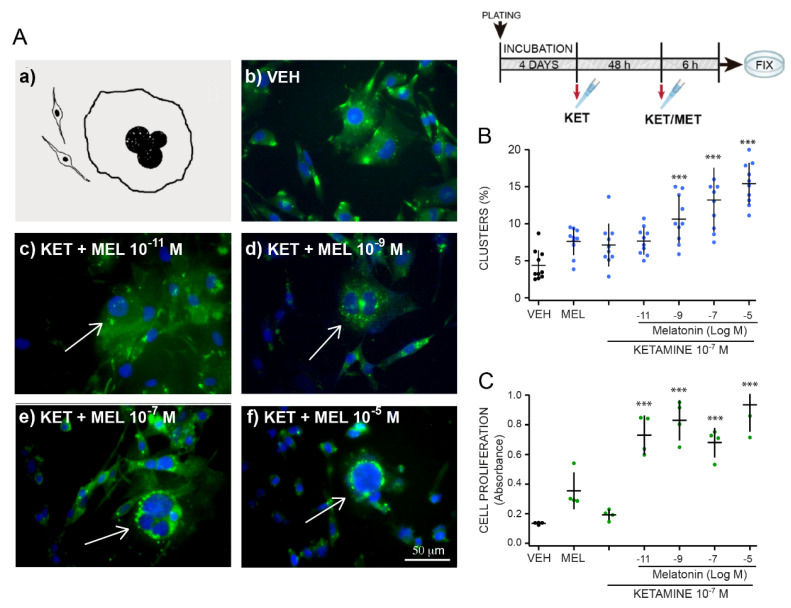
Concentration response effect of melatonin in combination with ketamine on formation of clusters in ONPs cultures pre-incubated for 48 h with ketamine. Olfactory neuronal precursors were cultured during 48 h with either the vehicle (VEH) or ketamine (KET 10^−7^ M) and then with KET (10^−7^ M) together with various MEL (10^−11^ to 10^−5^ M) concentrations. Cells were fixed and stained with an anti-nestin antibody followed by a secondary antibody coupled to FITC (green), and DAPI (blue). The inset to the right, above, shows the treatment scheme. Panel **A** shows a diagram of a typical cluster (**a**). Representative merged images of neuronal precursors incubated with the VEH (**b**) or KET 10^−7^ M for 48 h followed by incubation with the combination of KET 10^−7^ M with either 10^−11^ M (**c**), 10^−9^ M (**d**), 10^−7^ M (**e**) or 10^−5^ M (**f**) MEL. Bar = 50 μm. Clusters are pointed at using an arrow. Panel **B** shows the graph of the effect of melatonin on cluster formation in cells preincubated with 10^−7^ M KET for h. Clusters were counted in 10 fields in cultures treated as described and normalized by total number of cells in the field counted by DAPI staining (n = 10). Panel **C** shows a graph of the concentration–response curve of MEL effects on cell proliferation of ONPs cultured with KET 10^−7^ M determined by formazan formation. Each point represents the mean of absorbance values measured in 4 wells. The mean ± standard deviation of the mean (SEM) is from 1 or 2 experiments performed by quadruplicate. Each experiment was repeated three times. Data was analyzed by one-way ANOVA with Dunnett´s post-test. *** *p* = 0.001 when compared with vehicle (VEH) control.

Figure 3 shows the concentration response effects of MEL on spherical cluster formation in cells preincubated with 10^−7^ M KET. Representative images of clusters formed after 48 h incubation with KET and different concentrations of MEL for 6 h are shown in Figure 3A ok.

Increased concentrations of MEL caused an augmented number of cluster formation in ONPs previously challenged with KET 10^−7^ M for 48 h (Figure 3B). A concentration response effect was observed with increasing concentrations of MEL, and the number of clusters augmented three times in the presence of 10^−5^ M of the indolamine and KET 10^−7^ M (F_(4,45)_ = 21.53, *p* ≤ 0.001) (Figure 3B). Data were analyzed by the synergy finder software in R with the “zero interaction potency model” [30]. Results for MEL (10^−11^, 10^−9^, 10^−7^ and 10^−5^ M) and KET (10^−7^ M) showed the next scores: −9.19, −9.72, −10.32 y −11.85. Cell proliferation measured by formazan formation in cells treated with KET/MEL showed an increased formation of this compound regarding the cells incubated with the vehicle (Figure 3C) (F_(4,15)_ = 25.12, *p* ≤ 0.001). The data indicate that cluster formation is higher in the presence of MEL in combination with KET and suggest that the interaction of KET/MEL combination is additive.

### 2.4. The Anti-Depressant Schedule of KET/MEL Administration Increases Cluster Formation in Cultured Olfactory Neuronal Precursors

**Figure 4:**
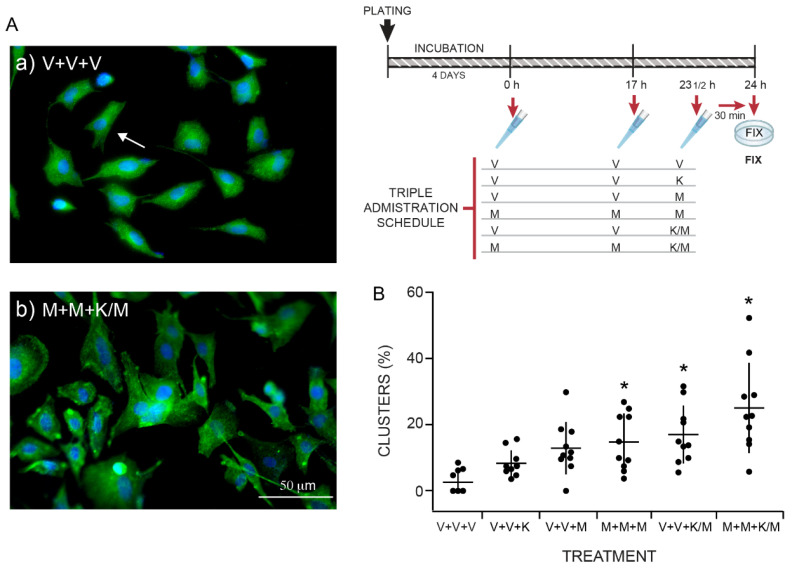
Incubation of ONPs with the triple administration schedule of melatonin/ketamine combination elicited cluster formation. Olfactory neuronal precursors were cultured with the vehicle added at time 0, 17 h and 23.5 h (V+V+V); or with VEH, VEH and KET; VEH, VEH, and MEL; MEL, MEL, and MEL; VEH, VEH, and KET/MEL or MEL, MEL, and KET/MEL combination (M+M+K/M). Panel **A** shows representative images of cells administered three times with the VEH (**a**) or with MEL+ MEL + KET/MEL combination (**b**). After fixation, cells were stained with DAPI (blue), and with an anti-nestin antibody (green). Single cells are noted using an arrow. Bar= 50 µm. Panel **B**: The graph shows the number of clusters counted in 10 fields in cultures treated as described and normalized by total cells in the field counted by DAPI staining. Each point in the graph represents the number of clusters counted in one field in the cultures incubated with the indicated treatments. Data are expressed as mean ± standard deviation obtained in ten fields. The experiment was repeated twice— and data were analyzed using a Kruskal–Wallis test followed by the Dunn´s post-test. * *p* = 0.007, when compared with the vehicle control group (V+V+V).

KET in combination with MEL was tested on cluster formation in cultured ONPs administered with the schedule we previously found to elicit an antidepressant-like effect and neurogenesis in mice [8]. The inset in Figure 4 shows the administration schedules. The cells were treated with a triple administration: The first administration (either the VEH or MEL) was given four days after plating (0 h). The second administration occurred 17 h after the first one (17 h), and the third administration (VEH, MEL, KET, or KET/MEL) occurred 6.5 h after the second one (23 ½ h). The cells were fixed 30 min after the third administration and stained with DAPI. Figure 4A shows images of clusters formed in cultures administered only with the VEH (a) or with MEL, MEL, KET/MEL (b). Quantitative data are shown in Figure 4B and cells cultured with KET/MEL (b) showed the maximal effect on cluster formation (H = 17.61, df = 6, *p* = 0.007). The results indicate that the schedule of administration that elicits the antidepressant-like effects and neurogenesis in mice also increases neurogenesis in cultures of ONPs. 

### 2.5. Comparison of KET/MEL Effects on Cluster Formation with Nerve Growth Factors 

**Figure 5:**
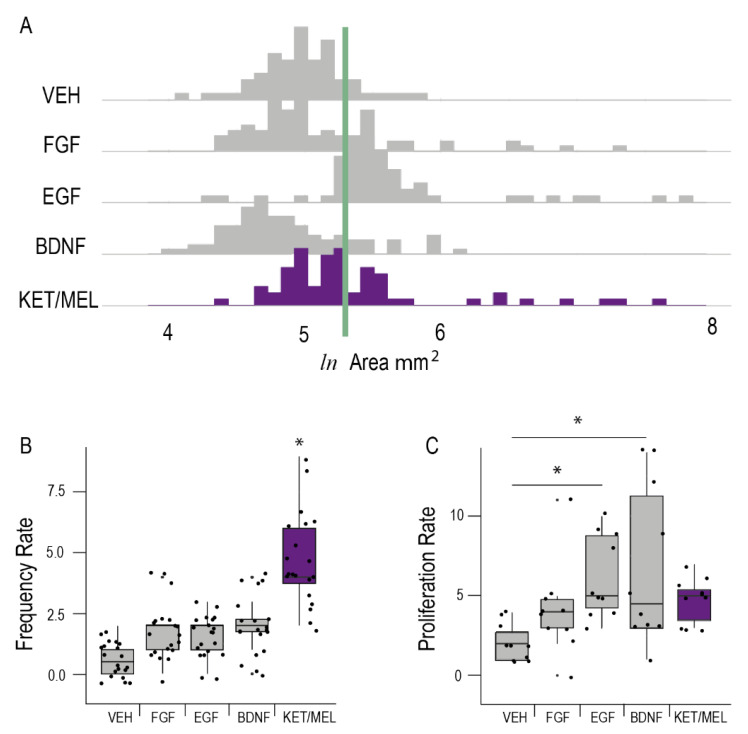
Comparison of the effects of KET/MEL combination with the effects of growth factors on neurogenesis. Olfactory neuronal precursors were cultured with the vehicle (VEH), fibroblastic growth factor (FGF), epidermal growth factor (EGF), brain-derived neurotrophic factor (BDNF) or the combination of ketamine and melatonin (KET/MEL) during 6 h. The cluster area (Panel **A**); the frequency of cluster formation (Panel **B**); and the proliferation rate (Panel **C**), were determined as described in the Materials and Methods section. The experiment was repeated twice, and data were analyzed using Kruskal–Wallis test followed by Dunn´s multiple comparison test. Results are expressed as median ± Tukey intervals. * Asterisks indicate significant differences with *p* < 0.05.

Figure 5 shows the area, the frequency of cluster formation and the proliferation rate of ONPs cultured during 6 h with the KET/MEL combination and in comparison, with FGF, EGF and BDNF. As shown in Figure 5A, a major number of clusters with large area was observed in cultures of ONPs incubated with the KET/MEL combination and EGF. Similarly, the frequency of cluster formation (Figure 5B) and the proliferation rate (Figure 5C) was higher in these cultures. However, BDNF had only a small effect in all these parameters. 

### 2.6. KN-62, Luzindole, and Trifluoperazine Inhibit Cluster Formation Elicited by KET/MEL Combination

**Figure 6:**
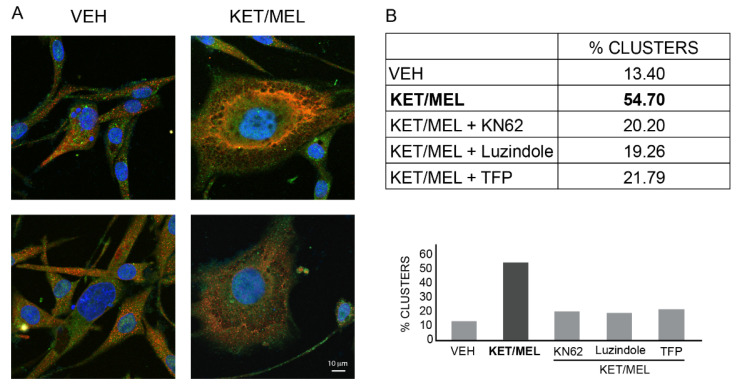
Participation of CaMKII, melatonin receptors, and calmodulin (CaM) on cluster formation. Olfactory neuronal precursors were cultured for 4 days and then preincubated in serum-free medium with the VEH, 10 µM KN-62, an inhibitor of CaM Kinase II, 10 µM luzindole, an MT1 and MT2 melatonin receptor antagonist, or 10 µM trifluoperazine (TFP), a CaM antagonist, during 15 min. Afterwards, the combination of 10^−7^ M ketamine with 10^−7^ M melatonin (KET/MEL) was added for 6 h. Cells were fixed and stained with an anti-nestin an anti-doublecortin antibodies, followed by secondary antibodies as described in Materials and Methods. Panel **A** shows representative images of clusters formed in the presence of the VEH or the KET/MEL combination. Bar = 10 µm. Panel **B** shows a table and a bar graph with the results of frequency formation of clusters. Results are the mean of 7000 cells/coverslip analyzed by duplicate per treatment (n = 4). Each coverslip has an area of 113 mm^2^ which nearly corresponds to 340 fields observed with the 40X objective. Coverslips were scanned by confocal microscopy with the Image J (NIH) and Zenblue (Zeiss) software. The experiment was repeated two times.

To explore the mechanism by which the KET/MEL combination elicits cluster formation, ONPs were preincubated with either the vehicle (VEH), KN-62 an inhibitor of CaMKII, luzindole a non-specific MT1 and MT2 melatonin receptor antagonist, or trifluoperazine (TFP) a CaM antagonist, followed by 6 h incubation with the KET/MEL combination. Figure 6A shows that clusters formed in the presence of KET/MEL are more complex than those formed with the VEH. Additionally, KN-62, luzindole or TFP inhibited nearly by 60% the frequency of cluster formation (Figure 6B), indicating that CaMK II, MEL receptors and CaM are involved in the signaling pathway by which KET/MEL combination cause cluster formation.

## 3. Discussion

In this study, we show that KET and MEL administered alone and in combination elicited neurogenesis in cultured ONPs, similarly to the nerve growth factors FGF, EFG and BDNF. Additionally, the KET/MEL combination administered at subeffective doses and with a schedule like that which produces antidepressant-like effects in mice, produced increased neurogenesis in cultured ONPs.

Previously, it was shown that neuronal precursors in culture form neurospheres derived from a single cell, that proliferate until being detached from the surface of the culture dish [10,14]. Here, we determined the number of clusters formed by ONPs at an early stage, which is when they still are adhered to the substratum. 

Previously, it was demonstrated that nestin is a marker for progenitor neurons, and doublecortin (DCX), a marker for newly formed neurons [28,29]. Additionally, the neurogenic nature of cells was demonstrated by BrdU incorporation into DNA and staining with an anti-BdrU antibody [6] and with an anti-Ki67 antibody, which label cells in proliferation [29,31] challenged with neuronal growth factors known to stimulate neurogenesis in neuronal cultures and in the dentate gyrus of the hippocampus [26,32,33,34]. 

Data obtained in our study showed that clusters of ONPs were stained with anti-nestin, anti-DCX, the markers of progenitor neuronal precursors and neurogenic neurons, respectively, as well as by anti-BrdU antibodies, or Ki67 which label proliferating cells [28,29]. Additionally, proliferation was measured by the amount of formazan formed in the presence of KET/MEL [35]. Thus, our data indicate that clusters are formed by proliferating neuronal precursors. However, some clusters are also formed by adhesion of neighboring cells, as demonstrated by the lack of Ki67 staining. This agrees with the recent concept published by Ladiwala et al., 2012, showing that clusters and neurospheres can also be formed by multiple cells that adhere together, and not only by a single cell that proliferates until forming a neurosphere [36]. Moreover, KET, MEL, or the KET/MEL combination increased the formation of clusters, indicating that these compounds stimulate neurogenesis similarly to the growth factors FGF, EFG, and BDNF. Thus, altogether data indicate that KET/MEL treatment induces neurogenesis in the in vitro cell culture model of human cloned ONPs.

Previously it was shown that BDNF, EFG and FGF growth factors induce antidepressant-like effects in mice [27,37]. Moreover, these growth factors stimulate neurogenesis in the dentate gyrus of the hippocampus which is presumably the way in which they produce antidepressant-like effects [24,32,33,34,38]. On the other hand, it has been suggested that the neuroepithelial tissue is a subrogate model to study molecular and metabolic changes produced in the central nervous system [12,39]. In this regard, our data support this concept, since growth factors were able to induce neurogenesis ex vivo in neuronal precursors derived from a human being, similarly to the reported effects on neuronal precursors derived from the subventricular zone of mice [34]. Thus, our data, together with the fact that these cells express receptors for dopamine, NMDA, BDNF, serotonin, among others [21], suggest that ONPs derived from neuropsychiatric patients can be challenged by different compounds in order to give a glimpse onto how the cells of any single patient respond to these treatments, and eventually this could lead to a preparation to test personalized pharmacological treatments.

To explore the ability of the KET/MEL combination to increase neurogenesis in cultured ONPs, we considered that anesthetic and psychodysleptic effects are produced in humans by KET at a range of 600–1200 ng/mL. Moreover, anxiety and paranoid feelings appear at 500 ng/mL [40]. Therefore, we devised a concentration–effect curve of KET on the human ONPs, using the range from anesthetic doses 3370 ng/mL (10^−5^ M) to 0.237 ng/mL (10^−11^ M), which reaches four orders of magnitude below the doses that cause anesthetic and psychedelic effects. Similarly, we tested physiological and pharmacological concentrations described for MEL in humans in plasma and in the cerebrospinal fluid during the night and after 10 mg of intravenous administration [41]. In our study, we found that cluster formation is elicited by KET at 100 nM, which is a concentration 2–3 orders of magnitude lower than anesthetic and psychomimetic doses. Similarly, we found MEL effects on neurogenesis at 100 nM, which is the concentration present in the cerebrospinal fluid at night [42]. Therefore, because the KET/MEL combination induces its neurogenic effects at nanomolar concentrations, which does not cause psychomimetic effects, it could be useful as a rescue therapeutic pharmacological treatment to those patients with depression resistant to treatment or those with suicidal ideations, because the antidepressant effects may be obtained in a therapeutically safe window, quickly and without collateral undesirable effects.

The mechanism by which the KET/MEL combination increases neurogenesis is not precisely known. KET is an antagonist of the NMDA receptor [43]. On the other hand, MEL stimulates MT_1_/MT_2_ receptors and in the rat *habenula* acts on the glutamatergic signaling system [44,45]. In addition, it has been reported that MEL increases BDNF levels [46]. In our study, cluster formation was inhibited by KN-62, luzindole and TFP, suggesting that CaMK II MEL receptors, as well as CaM, participate in the mechanism by which the combination stimulates neurogenesis. Previously, it was shown that MEL receptors participate in neurogenesis caused by MEL in mice [6,47]. On the other hand, NMDA antagonism by KET stimulates neurogenesis in cortical neurons [48]. Thus, altogether evidence suggest that the three types of receptors, MT1, MT2, and NMDA, may participate in the signaling pathway of the KET/MEL combination. In fact, MT1 and MT2 receptors are expressed in the clone of ONPs used in this study, and previously it was demonstrated that olfactory neurons express NMDA receptors [21,49]. On the other hand, mitochondria play a key role in proliferation and at mitochondrial level; melatonin maintains the membrane potential, ROS balance, and ATP synthesis, the intramitochondrial synthesis of melatonin, as well as the indolamine release in the cytosol and diffusion into the intracellular membranes where it can produce intracrine effects, especially in the binding to calmodulin and the activation of its protein targets such as CaMK II [50,51]. 

Despite this background knowledge, further research is necessary to explain in detail the mechanisms activated by the KET/MEL combination, as well as to disclose the molecules that participate in the signaling pathway downstream MEL and NMDA receptors. Finally, our results support that the ONPs derived from humans are a good translational model to study personalized pharmacological responses.

## 4. Materials and Methods

### 4.1. Cell Culture Protocol and Pharmacological Treatment

Olfactory neuronal precursors were obtained one time by nasal cavity exfoliation of the anterior region of the medial lateral turbinate from a human—a Latin American female participant of 54 years old. Cells were detached from the exfoliation brush by mechanical agitation and cultured to passage 4. Then, a clone was obtained by limiting dilution as described [52,53]. The experiments were carried out with the cloned cells for 20–30 passages.

ONPs were cultured in Dulbecco’s modified Eagle and F-12 media (DMEM/F-12) supplemented with 10% FBS, 4 mM L-glutamine, 100 μg/mL streptomycin, 100 IU/mL, penicillin, and 0.25 µg/mL amphotericin B. The clone was propagated, frozen, and stored in liquid nitrogen [52]. For the experiments, cells were replated at 10,000 cells/cm^2^ on 1.5 cm^2^ German round cover slips ((72196-12 Electron Microscopy Sciences, Hatfield, PA, USA) Germany), placed in 24-well multidishes (Corning Costar, Corning, NY, USA) and cultured for 4 days. Then, cells were incubated in serum free media with the vehicle (VEH) or the treatments. Cells were incubated with 20 ng/mL of either FGF, EGF or BDNF for 6 h. For concentration–response curves, ONPs were cultured with either KET or MEL at 10^−11^, 10^−9^, 10^−7^ or 10^−5^ M, and incubated for 6 h. After incubation, coverslips were washed tree times with PBS and cells fixed with 4% paraformaldehyde for 20 min and stored at 4 °C until their use. 

For the experiments shown in Figure 3, cells were treated with 10^−7^ M KET for 48 h followed by 6 h incubation with of 10^−11^, 10^−9^, 10^−7^, or 10^−5^ M MEL. In other experiments (Figure 4), ONPs were treated using a triple administration schedule. Cells were treated with the VEH or 10^−7^ M of MEL at time 0 and 17 h. The 3rd administration was MEL (10^−7^ M), KET (10^−7^ M), or the combination, 6.5 h after the second one, and 30 min before fixation. For the experiments with pharmacological inhibitors (Figure 6), cells were treated with KN-62 (10 µM), luzindole (10 µM) or TFP (10 µM) for 15 min before adding KET/MEL 10^−7^ M. Cells were further incubated for 6 h before fixation. MEL and KET were dissolved in 60 µL of ethanol and 940 µL DMEM, respectively, to prepare a 1 mM stock solution. The final ethanol concentration was 0.06% in the culture media. 

### 4.2. Immunofluorescence Staining

Cultures were processed for immunofluorescence as described [52]. Neuronal precursors and clusters were stained with an anti-nestin antibody (MAB5326, Millipore, Burlington, MA, USA) at 1:50 dilution and anti-doublecortin (ab207175, Abcam, Cambridge, UK) at 1:50 dilution. Proliferating cells were in some cases preloaded with 10 µM BdrU for 6 h and then recognized with an antibody raised against BrdU (1:100; ab8152 Abcam, Cambridge, UK) or with an anti-Ki67 (ab15580, Abcam, Cambridge, UK) at a 1:50 dilution [14]. Secondary antibodies were tetramethylrhodamine isothiocyanate (TRITC) coupled anti-mouse antibody (115 025 003, Jackson Immunoresearch, West Grove, PA, USA) in a dilution of 2:100 that recognizes the anti-nestin antibody and an anti-rabbit antibody coupled to FITC (11-095-152 Jackson Immunoresearch, West Grove, PA, USA), diluted 1:50, that recognized the anti-doublecortin antibody. Nuclei were stained with 300 nM 4′,6-diamidino-2-phenylindole (DAPI) (D1306Thermo Fisher Scientific, Waltham, MA, USA) and actin microfilaments with 200 nM phalloidin coupled to TRITC (phalloidin-TRITC) (Molecular Probes, Eugene, OR, USA) for 30 min at room temperature as described [17]. Coverslips were mounted in Vectashield (D1306, Thermo Fisher Scientific, Waltham, MA, USA). 

For the experiments in Figure 6, the images were taken with an inverted confocal microscope, the LSM900 Carl Zeiss (Oberkochen, Germany). The acquisition of images was made in Sample Navigator mode with a 10× objective. This mode allowed us to obtain images of the entire area of the coverslip. For the rest of experiments, cells and clusters were observed with an Inverted Eclipse TE 2000 microscope. Images were acquired with a digital sight camara, the DS-2MV (NIKON, Melville, NY, USA), as previously described [49]. Proliferating clusters were counted in each of 10 fields and were normalized by the total number of cells determined by DAPI staining in each field. In other experiments, the cluster area was measured by using the NIS ELEMENTS DS3 software from NIKON (NIKON, Melville, NY, USA).

For each pharmacological treatment, the images were analyzed using the open software Image J (NIH), with the implementation of semi-automatic Macros (Instruction written in a program language that is equivalent to a sequence of actions) that allowed us to determine the areas of the regions of interest (ROI). The data of the areas to be acquired from the binary mask of each sample navigator coverslip image were processed and analyzed using the macros implementation in Visual Basic Excel programming language. Clusters are considered areas over of 200 mm^2^ (≈50 mm cluster diameter).

### 4.3. Cell Proliferation Assay

Neuronal precursor´s proliferation was measured by the cleavage of tetrazolium WST1 to form the colored compound formazan with a commercial kit (Chemicon International). Absorbance was measured at 440 nm in a microplate reader (Coulter DTX 880 from Beckman) [35]. 

### 4.4. Statistical Analysis

Data that met the criteria of normality (Kolmogorov–Smirnov test) and variance equality were compared using a One-Way Analysis of Variance (ANOVA). Dunnett´s or Bonferroni´s tests for multiple comparisons vs. control group were applied when the ANOVA showed significant difference; *p*-values ≤ 0.05 were considered to be statistically significant. When data did not meet normality or variance equality criteria, a non-parametric analysis Kruskal–Wallis analysis of variance on ranks (* *p* ≤ 0.05; ** *p* ≤ 0.01; and *** *p* ≤ 0.001) was used, followed by the Dunn´s multiple comparison test. Data and statistical analysis were carried out using the Sigma Plot Program (version 12.3), R software (Version 4.1.3), R-Studio (Version 2022.02.3 ₊ 492). Groups analyzed by ANOVA or Kruskal–Wallis were unbalanced. Graphics were performed with the GraphPad Prisma 7 Software. A concentration–dose response curve for the combination was analyzed by the synergyfinder software in R with the “zero interaction potency” model [30].

## 5. Conclusions

Our results show that FGF, EGF, BDNF, KET, MEL and the KET/MEL combination stimulate neurogenesis and cell cluster formation in cultured ONPs derived from a living human, thus shedding light on the mechanism for producing antidepressant effects. Our results further indicate that cultured ONPs are a useful translational model to study biological responses elicited by antidepressant drugs, neuronal growth factors and other drugs, which could be used in determining personalized medical treatments for neuropsychiatric disease.

## 6. Patents

G.B.-K., R.E.-R., M.L.D., C.T. and D.B.Q.-C. are co-inventors on a patent for the use of the ketamine and melatonin combination in the treatment of mood disorders and assigned their patent’s rights to the Instituto Nacional de Psiquiatría Ramón de la Fuente Muñiz; they will share a percentage of any royalties that may be received by the Institution. 

## Data Availability

The data presented in this study are available on request from the corresponding author.

## Sample Availability

Cloned ONPs are available from GBK.
